# Supplementary material for: Cloud BioLinux: pre-configured and on-demand bioinformatics computing for the genomics community
Source: BMC Bioinformatics. 2012 Mar 19;13:42. doi: 10.1186/1471-2105-13-42 (PMC3372431; doi:10.1186/1471-2105-13-42)
Supplement: Additional file 1 — Supplementary 1 Cloud BioLinux software documentation in the form of a mini, self-contained website. Users need to download and uncompress the .zip file, and open through a web browser the "index.html" file available on the main directory. (ZIP 1823 kb). [file 1471-2105-13-42-S1.ZIP › Cloud-BioLinux-Package-Documentation/Display.html]

Bio-Linux Software Documentation Pages

Back to search form

### Display

|  |  |
| --- | --- |
| Category | Applications |
| Display | cytoscape    showpep    cn3d    textsearch    sixpack    showseq    showdb    seealso    remap    prettyseq    pepwheel    pepnet    lindna    cirdna    abiview    trev |
| SubCategory | Applications |
| Alignments | boxshade    jprofilegrid |
| Annotation | act    artemis    gff2ps    showfeat |
| Dotplots | dotter    lalign    lav2ps    lav2svg |
| Sequence\_traces | consed    gap4    trev |
| Structure | cn3d    rasmol |
